# Novel Salicylic Acid Analogs Induce a Potent Defense Response in Arabidopsis

**DOI:** 10.3390/ijms20133356

**Published:** 2019-07-08

**Authors:** Ian Arthur Palmer, Huan Chen, Jian Chen, Ming Chang, Min Li, Fengquan Liu, Zheng Qing Fu

**Affiliations:** 1 Department of Biological Sciences, University of South Carolina, Columbia, SC 29208, USA; 2 Institute of Plant Protection, Jiangsu Academy of Agricultural Sciences, Jiangsu Key Laboratory for Food Quality and Safety-State Key Laboratory Cultivation Base of Ministry of Science and Technology, Nanjing 210014, China

**Keywords:** salicylic acid, analogs, NPR1, NPR3, NPR4, PR1, *Pseudomonas syringae*

## Abstract

The master regulator of salicylic acid (SA)-mediated plant defense, NPR1 (NONEXPRESSER OF PR GENES 1) and its paralogs NPR3 and NPR4, act as SA receptors. After the perception of a pathogen, plant cells produce SA in the chloroplast. In the presence of SA, NPR1 protein is reduced from oligomers to monomers, and translocated into the nucleus. There, NPR1 binds to TGA, TCP, and WRKY transcription factors to induce expression of plant defense genes. A list of compounds structurally similar to SA was generated using ChemMine Tools and its Clustering Toolbox. Several of these analogs can induce SA-mediated defense and inhibit growth of *Pseudomonas syringae* in *Arabidopsis*. These analogs, when sprayed on *Arabidopsis*, can induce the accumulation of the master regulator of plant defense NPR1. In a yeast two-hybrid system, these analogs can strengthen the interactions among NPR proteins. We demonstrated that these analogs can induce the expression of the defense marker gene *PR1*. Furthermore, we hypothesized that these SA analogs could be potent tools against the citrus greening pathogen *Candidatus liberibacter* spp. In fact, our results suggest that the SA analogs we tested using *Arabidopsis* may also be effective for inducing a defense response in citrus. Several SA analogs consistently strengthened the interactions between citrus NPR1 and NPR3 proteins in a yeast two-hybrid system. In future assays, we plan to test whether these analogs avoid degradation by SA hydroxylases from plant pathogens. In future assays, we plan to test whether these analogs avoid degradation by SA hydroxylases from plant pathogens.

## 1. Introduction

Plant immunity can be described as consisting of four phases, known as the zig-zag model [1]. First, pathogen-associated molecular patterns (PAMPs) are recognized by pattern recognition receptors (PRRs) on the plant cell’s surface. Pathogen-associated molecular patterns are evolutionarily conserved molecules associated with pathogens such as flagellin, EF-Tu, and chitin [2,3,4]. Pathogen-associated molecular pattern recognition results in PAMP-triggered immunity (PTI). PAMP-triggered immunity consists of an increase in cytosolic Ca^2+^ [5], oxidative burst [6], MAPK activation [7], ethylene production [8], stomatal closure, transcriptional reprogramming, accumulation of the plant defense hormone salicylic acid (SA) [9], and callose deposition [10]. This response is basal disease resistance against pathogens that can halt colonization. During the second phase of the zig-zag model, pathogens deliver effectors into plant cells to interfere with PTI, resulting in effector-triggered susceptibility (ETS). Plants have evolved rsistance (R) proteins capable of specifically recognizing secreted effectors, resulting in effector-triggered immunity (ETI), as phase three. Resistance proteins are nucleotide-binding leucine-rich repeat (NB-LRR) proteins that can respond to effectors from plant pathogens [11,12]. Resistance proteins usually recognize effectors indirectly. They may act as accessory recognition proteins that detect effector modification of the effector’s true virulence targets or decoys that mimic the effector’s targets [13]. In phase four, pathogens either lose effector genes or acquire additional effector genes that can continue to suppress ETI and PTI. The loss of recognized effectors or the gain of novel effectors, causes selective pressure on the host to evolve new R proteins, resulting in ETI [1].

SA acts as a major plant hormone, playing a regulatory role in various physiological processes. These processes are diverse, and include seed germination, storage, fruit maturity, regulation of flower development, sex differentiation, stomatal movement, and photoperiod [14]. SA is necessary to induce a defense response against pathogens [14] and exogenous application of SA is sufficient to induce a defense response [15]. Transgenic *Arabidopsis* plants expressing *NahG* from *Pseudomonas putida*, encoding a SA hydroxylase enzyme which degrades SA into catechol, are rendered more susceptible to a variety of pathogens [16].

SA is synthesized in the chloroplast after pathogen perception. In *Arabidopsis*, SA biosynthesis is produced primarily through the isochorismate pathway, in which chorismate is converted into isochorismate by ICS1 (isochorismate synthase 1) and then isochorismate is presumably converted into SA by an unidentified IPL (isochorismate pyruvate lyase) [17]. Isochorismate synthase 1 is localized in the plastid and is responsible for the majority of SA accumulation in response to the presence of hemibiotrophic and biotrophic pathogens [18,19]. The *Arabidopsis ics1* mutants are unable to accumulate SA, and consequently, *ics1* mutants are more susceptible to pathogen infection. SA can induce a potent systemic immune response known as systemic acquired resistance (SAR) [20].

The NPR1 and NPR1 paralogs NPR3 and NPR4 bind SA and function as SA receptors [21,22,23]. NPR1 functions as a transcriptional co-activator. Upon pathogen infection, NPR1 oligomers in the cytosol are reduced into monomers and then NPR1 monomers enter the nucleus and interact with TGA and TCP transcription factor to activate the expression of *PR* genes, which encode small proteins that may have antimicrobial properties [24,25,26]. Induction of the expression of *PR1* is directly correlated with an increase of SA levels [27]. The NPR1 paralogs NPR3 and NPR4 act as adaptor proteins for Cullin 3 E3 ubiquitin ligase, leading to the ubiquitination and degradation of NPR1, dependent on SA concentration—a high level of SA disrupts the interaction between NPR1 and NPR4, while promoting the interaction between NPR1 and NPR3; this creates a biphasic pattern of NPR1 level and defense response [28]. Both NPR3 and NPR4 are also known to form homo- and heterodimers and the formation of NPR3 and NPR4 homo- and heterodimers is strengthened by the presence of SA [21].

In addition to inducing a local defense response, SA promotes systemic acquired resistance (SAR) after an invading pathogen is recognized [29,30]. Systemic acquired resistance protects the plant against further pathogen colonization by causing a systemic defense reaction including the production of pathogenesis related (PR) proteins, phytoalexins, and the strengthening of cell walls. SA is also responsible for regulating these later responses to pathogenic invasion [31], and application of SA is sufficient to induce plant defense including SAR [32].

**Figure 1:**
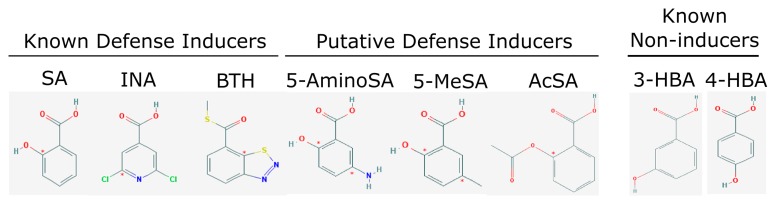
Comparison of known defense inducers and known non-inducers. Known inducers often have substitutions on carbon two and/or carbon five of the aromatic ring. Non-inducers have substitutions on carbon three or four. Substitutions on carbons two or five are indicated by a red asterisk. SA is salicylic acid, INA is 2,6-dichloroisonicotinic acid, BTH is benzothiadiazole, 5-AminoSA is 5-aminosalicylic acid, 5-MeSA is 5-methylsalicylic acid, AcSA is acetylsalicylic acid, 3-HBA is 3-hydroxybenzoic acid, and 4-HBA is 4-hydroxybenzoic acid.

The SA-mediated plant defense pathway can be activated by exogenous application of SA, 2,6-dichloroisonicotinic acid (INA), or benzothiadiazole (BTH) (Figure 1) [33,34]. Additionally, some synthetic compounds have been used in the past to elicit a defense response, protecting crops from disease. These synthetic compounds include 3-allyloxy-1,2-benzisothiazole-1,1-dioxide (probenazole, PBZ), applied to *Oryza sativa* to prevent rice blast caused by *Magnaporthea grisea* [35]; the previously mentioned INA on *Cucumis sativus* and *Nicotiana tabacum* to prevent anthracnose (caused by *Colletotrichum lagenarium*) and tobacco mosaic virus infection, respectively [15,36]; N-cyanomethyl-2-chloroisonicotinamide (NCI) on *O. sativa* to induce defense against *Pyricularia oryzae*, an anamorph of *M. oryzae*, and many others [37,38].

Unsurprisingly, due to the necessity of SA for defense induction, pathogens have evolved enzymes capable of degrading this key phytohormone. Bacterial members of the genera *Pseudomonas, Bacillus, Agrobacterium, Rhizobium, Sinorhizobium, Ralstonia,* and *Burkholderia* have genes encoding SA hydroxylases capable of metabolizing SA into less active forms [39]. SA hydroxylases function typically by binding SA and NADH or NADPH, then binding molecular oxygen. The resulting products are catechol, H_2_O, and CO_2_ [40]. Ectopically expressing the bacterial SA hydroxylase gene, *NahG*, from *Pseudomonas putida* in *Arabidopsis* suppresses the defense response against both bacterial and fungal pathogens and abolishes SA accumulation after pathogen infection [41].

Here, we present the results of a screen of SA analogs. We demonstrate that by applying several of these analogs to *Arabidopsis* Col-0 plants, the accumulation of the master regulator of SA-mediated plant defense, NPR1, can be induced. We show that the application of these SA analogs results in the induction of *PR1* expression. We demonstrate that these SA analogs can strengthen the protein–protein interactions between NPR1 paralogs NPR3 and NPR4 in a yeast two-hybrid system. We demonstrate that these analogs are effective in inhibiting bacterial growth, causing increased resistance against pathogen infection. We also demonstrate that a similar group of SA analogs that are functional in *Arabidopsis* are also capable of strengthening the interactions between NPR1 and NPR3 homologs in *Citrus sinensis.*

## 2. Results

### 2.1. ChemMine Results

The simplified molecular-input line-entry system **(**SMILES) string for SA, c1ccc(c(c1)C(=O)O)O, was used as input for ChemMine Tools. This online suite of tools allows for comparing pairwise structural similarities between compounds and provides ultra-fast structure similarity search algorithms. ChemMine Tools also contains a Clustering Toolbox to group the mined chemicals based on systematic structure and predicted activity [42]. This suite of tools was used to find the 50 most similar compounds to SA, compiled into an excel workbook. Candidate chemical compounds were then sorted by logP value and eliminated from the list based on predicted logP value (Table 1).

Of the list of 50 most similar compounds to SA, we selected compounds that had substitutions on the second or fifth carbon of the six-carbon ring. We hypothesized that substitutions made on the second or fifth carbon may be key to developing novel SA analogs that are functional but may resist degradation by bacterial pathogens (Figure 1), based on a comparison of the molecular structures of known defense inducers, SA, INA, and BTH, compared with known non-inducers, 3-hydroxybenzoic acid (3-HBA) and 4-hydroxybenzoic acid (4-HBA). The complete list of SA analogs tested in this work and in Figure 1 can be found in Table 2. In this paper, we focused on sodium salicylate (NaSA) as a positive control. Sodium salicylate is a water-soluble form of SA that dissociates, forming SA in solution. Ethyl salicylate (EtSA), acetylsalicylate (ACSA), 5-methylsalicylic acid (5-MeSA), 5-aminosalicylic acid (5-amino-SA) or mesalamine, 5-fluoro-2-hydroxybenzoic acid (5-F-2-HBA), and 5-iodosalicylic acid (5-I-SA), 2HTPA (2-hydroxyterephthalic acid), 2,4-dihydroxybenzoic acid (2, 4DHBA), 2,5-dihydroxybenzoic acid (2,5 DHBA), and 4HBA (4-hydroxyl-benzoic acid) as a negative control.

### 2.2. Several Putative SA Analogs Increased the Strength of Interactions among NPR3/4 in Y2H

**Figure 2:**
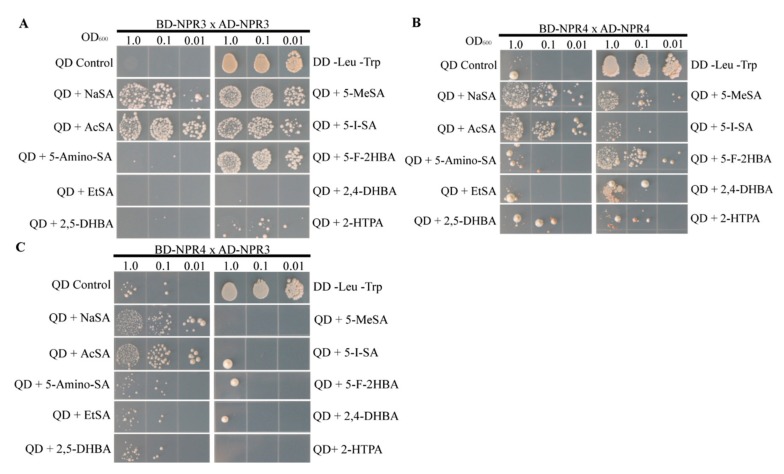
Several SA analogs consistently strengthened the interactions among NPR proteins in a Y2H system. (**A**) Interaction between NPR3 and NPR3; (**B**) interaction between NPR4 and NPR4; (**C**) interaction between NPR4 and NPR3. Yeast strains were incubated for 24 h in double dropout liquid media before being washed in sterile deionized water, diluted, and plated on quadruple dropout agar media with or without 200 μM NaSA or SA analogs. Plates were incubated at 30 °C for 72 h. QD is quadruple dropout –Leu–Trp–His–Ade. DD is double dropout –Leu–Trp. The assay was repeated three times with similar results.

Due to the critical role that NPR1 paralogs NPR3 and NPR4 play in SA-mediated defense, we hypothesized that active SA analogs would increase the strength of the interactions among these proteins in a yeast two-hybrid system. Because the interaction between NPR1 and NPR3 is strengthened in response to SA and the interaction between NPR1 and NPR4 is disrupted by SA, we chose to examine the effects of SA analogs on the NPR3 and NPR4 interactions, which are strengthened by the presence of SA [21]. By examining the interactions between NPR1 paralogs instead of NPR1 itself, we hoped to remove some ambiguity from our Y2H results, resulting from the SA analogs both strengthening and disrupting interactions between NPR1 and its paralogs in Y2H. Indeed, we observed that several SA analogs cause an increase in the number of yeast colonies that survive on quadruple dropout media. The number of surviving colonies treated with SA analogs can be compared to the number that grow when treated with sodium salicylate, appearing when diluted to OD_600_ 0.01. As shown in Figure 2, we observed that 5-meSA and 5-F-2HBA can strengthen the interaction between NPR3 with NPR3 and NPR4 with NPR4. 5-I-SA can strengthen the interaction between NPR3 and NPR3. AcSA can strengthen the interaction between both NPR3/4 homo- and heterodimers.

### 2.3. Several SA Analogs Induced NPR1 Accumulation

**Figure 3:**
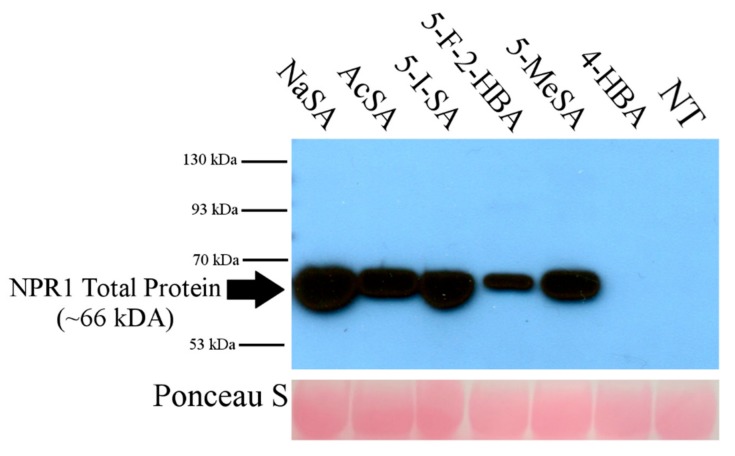
SA analog treatment induces accumulation of NPR1. Three-week-old *Arabidopsis thaliana* were sprayed with 1 mM NaSA or SA analogs. Samples were collected 6 hpi. Composite samples were taken consisting of one leaf each of a similar size and age from four plants, and 100 μg of protein was electrophoresed per sample. The membrane was incubated with anti-NPR1 antibody overnight at 4 °C. NT is non-treated. The assay was repeated three times with similar results.

Next, to determine whether these SA analogs could induce the accumulation of NPR1, we treated wild-type *Arabidopsis* with a 1 mM spray of SA analogs or SA, and compared the NPR1 protein levels, using untreated plants as a negative control. Previous research has shown that exogenous application of SA is sufficient to elicit a defense response, including the accumulation of NPR1. As shown in Figure 3, we found that AcSA, 5-I-SA, 5-F-2HBA, and 5-MeSA can induce NPR1 accumulation. 4-HBA and non-treated plants were included as negative controls. NaSA was included as a positive control.

### 2.4. Several SA Analogs Inhibited Bacterial Growth

**Figure 4:**
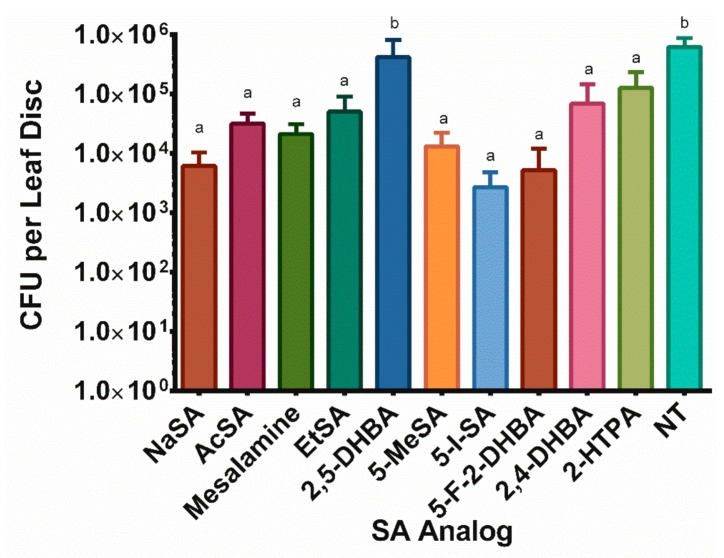
SA analog treatment reduced the number of bacteria present in leaves of treated plants. Three-week old *A. thaliana* Col-0 were sprayed with 1 mM NaSA or SA analogs. After 24 h, two leaves each from three plants per treatment were infiltrated with *Pseudomonas syringae* pv. maculicula ES4326 at OD600 0.001 in 10 mM MgSO4. After 72 h, 2 discs were sampled from each leaf. Dunnett’s multiple comparison test was used to generate groups of statistical significance. *p* ≤ 0.05. NT is non-treated. The assay was performed twice with similar results.

After observing that SA analogs could induce the accumulation of NPR1 in planta, we were curious whether treatment with SA analogs could inhibit the growth of plant bacterial pathogens. We observed that all but one SA analog, 2,5-DHBA, could reduce the number of CFUs per leaf disc by at least one order of magnitude, when compared with non-treated plants (Figure 4). Additionally, we observed no significant difference between the number of bacteria found in the SA-analog-treated plants and the SA-treated plants, again with the exception of 2,5-DHBA.

### 2.5. SA Analogs that Induced NPR1 Accumulation Were Inducers of *PR1* Expression

**Figure 5:**
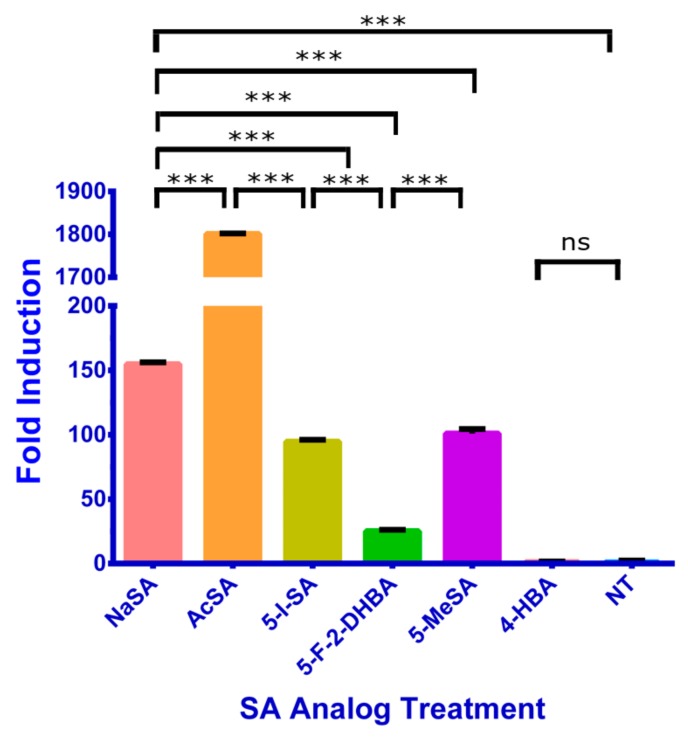
Relative normalized *PR1* expression 24 h after 1 mM SA analog spray. Composite samples were made from five biological replicates. Samples were assayed using three technical replicates. Expression levels were calculated using the double-delta Ct method. Error bars represent standard error of measurement. Expression levels of *NPR1* were normalized to the expression levels of *Ubiquitin 5* (*UBQ5*) (∆Ct ANOVA *p* < 0.0001; Student’s *t*-test *** *p* < 0.0001; ns is no significance).

After observing that almost all SA analogs could inhibit the growth of *P. syringae* and that several analogs were potent inducers of NPR1 accumulation, we hypothesized that an increase in NPR1 protein must trigger the expression of *PR1*, a gene encoding a small peptide which is known to inhibit the growth of bacterial pathogens. We sprayed Col-0 *Arabidopsis* with 1 mM SA or SA analogs, then collected leaf samples for RT-qPCR after 24 h. We observed that AcSA induces the highest level of *PR1* accumulation, even higher than the same concentration of NaSA. we observed that several other SA analogs could induce *PR1* expression, but at lower levels than NaSA or AcSA (Figure 5).

### 2.6. The Interaction Between CsNPR1 and CsNPR3 Was Strengthened by Several SA Analogs

**Figure 6:**
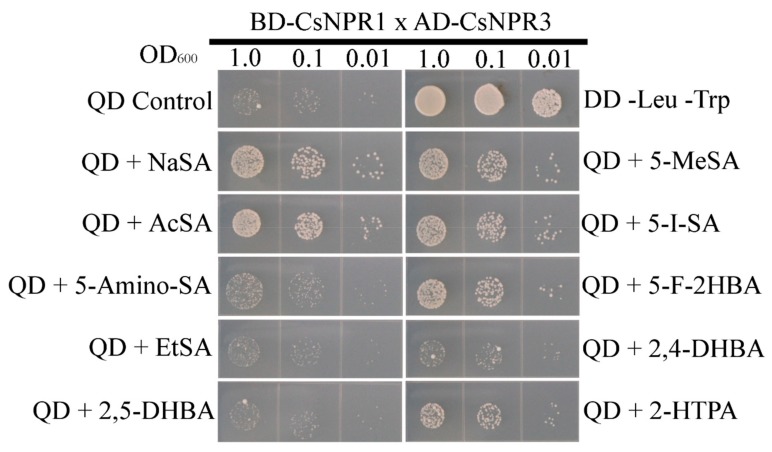
Several SA analogs consistently strengthen the interactions between citrus NPR1 and NPR3 proteins in a Y2H system. Yeast strains were incubated for 24 h in double dropout liquid media before being washed in sterile deionized water, diluted, and plated on quadruple dropout agar media with or without 200 μM SA or SA analogs. Plates were incubated at 30 °C for 72 h. QD is quadruple dropout –Leu–Trp–His–Ade. DD is double dropout–Leu–Trp.

We hypothesized that these SA analogs could be potent tools against the citrus greening pathogen, *Candidatus liberibacter* spp., which is known to produce an SA hydroxylase enzyme that functions to suppress plant defense [39]. We cloned the NPR1 and NPR3 homologs from *Citrus sinensis* Valencia and tested whether the SA analogs could also strengthen the interaction among citrus NPR proteins using Y2H (Figure 6). The NPR homologs have been identified previously in citrus [44]. The citrus NPR1 homolog CtNH1 (Citrus NPR1 homolog 1) has been previously shown to induce *PR* gene expression in *Citrus maxima* and confer resistance to the bacterial pathogen *Xanthomonas axonopodis* pv. *citri* [44]. We observed that NaSA, AcSA, 5-MeSA, 5-we-SA, 5-F-2-HBA, and 2-HTPA all can strengthen the interaction among citrus NPR proteins in our Y2H system. This finding is significant, because it suggests that the SA analogs we tested using *Arabidopsis* may also be effective for inducing a defense response in citrus. If these SA analogs are active in citrus, then we speculate that they may be candidates for fighting the citrus greening pathogen, because they may not be able to be degraded by the pathogen’s SA hydroxylase enzyme.

## 3. Discussion

In this study, we found acetylsalicylate, 5-methylsalicylic acid, 5-fluoro-2-hydroxybenzoic acid, and 5-iodosalicylic acid to be reliable inducers of plant defense. The data we have presented here suggest that these SA analogs would be worthy candidates for use against bacterial pathogens. Their ability to invoke a defense response from *Arabidopsis* and confer bacterial resistance are traits that warrant further investigation.

Previous research suggested that acetylsalicylate was effective against tobacco mosaic virus in tobacco [45]; however, there is little research into its use against bacterial pathogens. Acetylsalicylate’s ability to induce defense is not entirely surprising when one considers that acetylsalicylic acid and SA also share a function in mammals. The ability for acetylsalicylate to induce a higher level of PR1 accumulation and *PR1* expression may be due to an increase in membrane permeability of that compound in relation to sodium salicylate. A compound’s polar surface area can be used as a measure of that compound’s H-bonding potential, and therefore, its membrane penetration potential [46]. Acetylsalicylate has a slightly higher polar surface area at 63.6 Å^2^ than sodium salicylate which is 60.4 Å^2^ [47], which could make it slightly more bioavailable to the treated plant’s cells.

5-Fluoro-2-hydroxybenzoic acid and 5-iodosalicylic acid are likely inducers of plant defense because of their structural similarity to SA. Usually, the chemical interaction between a protein and a small molecule is dictated by electrostatic forces—H-bonding and Van der Waals forces—but halogen atoms can also generate intermolecular forces capable of stabilizing a protein complex that are similar to H-bonding in both strength and directionality [48]. This realization has enabled researchers to develop new halogen-substituted ligands that are more membrane permeable and have a longer biological half-life by avoiding the normal catabolic processes that normally degrade the drug [48]. For these reasons, 5-F-2HBA and 5-we-SA would make great candidates for use against pathogens that produce SA hydroxylase enzymes.

Previous research has suggested that SA acts directly on *P. syringae*, acting as an anti-microbial agent and reducing biofilm formation [49]. In addition to their ability to induce plant defense, these SA analogs may also act directly to inhibit the growth of *P. syringae* and other pathogens. As demonstrated in Figure 4, all SA analogs, except for 2,5-DHBA, reduced the bioburden of *P. syringae* after treatment; however, more research is needed to demonstrate the direct action of these SA analogs on pathogen growth and biofilm formation.

Our research demonstrates that 5-methylsalicylic acid can induce NPR1 accumulation, *PR1* expression, inhibit pathogen growth, and promote the interaction between NPR proteins. 5-MeSA differs from methyl salicylate (MeSA), which has a methyl group appended to the carboxyl group on carbon 1 of the aromatic ring, rather than the methyl substitution on carbon 5. Unlike methyl salicylate which is a volatile, wintergreen-scented compound that is a liquid at room temperature, 5-MeSA is a white, odorless compound that is solid at room temperature. 5-MeSA’s use as a defense inducer warrants further research, because it is similar enough in structure to SA, but may be able to avoid degradation by bacterial SA hydroxylases due to the methyl group substitution on carbon 5.

Currently, INA and BTH are widely used as active salicylic acid analogs. However, both of these active analogs have major drawbacks. Because of its toxic side effects, INA has never been commercialized [38]. BTH has been commercialized as Bion^®^ or Actigard^®^ but is very expensive because the complex structure is costly to synthesize. Our newly identified salicylic acid analogs could potentially be used to replace INA and BTH for controlling plant diseases.

## 4. Materials and Methods

### 4.1. Yeast Two-Hybrid (Y2H) Assays

Yeast strains were mated in yeast extract, peptone, dextrose, adenine (YPDA) media for 48 h at 30 °C. Diploid yeast strains were plated on double dropout selective media. Colonies were selected, then grown for 48 h in liquid double dropout media at 30 °C. The resulting liquid culture was serially diluted to an OD600 value of 1.0, 0.1, and 0.01, then plated on quadruple synthetic dropout media with and without SA or SA analogs and incubated at 30 °C for 72 h. CsNPR1 and CsNPR3 were cloned from *Citrus sinensis* Valencia into pDONR^®^ 207 using the Gateway BP reaction. The Gateway LR reaction was used to generate pGADT7 and pGBKT7 yeast expression vectors containing CsNPR1 or CsNPR3. These vectors were transformed into yeast strains Y187 or AH109, respectively, then the yeast strains were mated and plated on synthetic quadruple dropout (QD) media with and without SA or SA analogs like the previously conducted Y2H assays. The yeast strains expressing *Arabidopsis* NPR1, NPR3, and NPR4 were described in previous works [21]. QD agar lacking SA or SA analogs was used a negative control for Y2H, because the NPR protein interactions were previously described using the same Y2H system.

### 4.2. SA Analog Spray Treatment

SA analogs were diluted in 50 mL sterile purified water to a final concentration of 1 mM. The SA analog solutions were sprayed using a Preval^®^ Sprayer. The *Arabidopsis* leaves were sprayed from multiple angles until the leaves were visibly wet to ensure complete coverage. Between applications, the Preval^®^ Sprayer was washed, and 15 mL of sterile purified water was sprayed through to ensure no cross contamination of SA analogs.

### 4.3. Immunoblotting

Three-week-old *Arabidopsis thaliana* plants were sprayed with 1 mM SA or SA analogs as above. Samples were collected 6 h after treatment for assaying NPR1 accumulation. Composite samples were taken consisting of one leaf each of a similar size and age from four plants. Leaves were frozen in liquid nitrogen, then ground using a metal bead by crushing for 2 min at 1200 RPM. Protein was extracted using 1× protein extraction buffer (50 mM Tris-HCL, pH 7.5, 150 mM NaCl, 5 mM EDTA, 0.1% Triton X-100, 0.2% IGEPAL CA-630) with 1× protease inhibitor cocktail (Millipore Sigma, Burlington, MA, USA), 10 mM DTT, 1 mM PMSF, and 10 mM MG115. Samples were centrifuged at 15,000× *g* for 30 min at 4 °C and the supernatant removed to a new tube. The centrifugation was repeated twice. The protein concentration was determined by mixing 5 μL of protein sample with 200 μL of 5× Bradford reagent (Bio-Rad, Hercules, CA, USA) in a spectrophotometer cuvette and filling to 1 mL with sterile deionized water. The samples were analyzed for absorbance at 595 nm. Protein concentration was determined by comparing the absorbance to a standard curve. One-hundred micrograms of protein were boiled for 10 min in 1× Laemmli sample buffer (2% *w*/*v* SDS, 10% Glycerol, 60 mM Tris-HCL pH 6.8, 0.01% bromophenol blue, 0.2% 2-mercaptoethanol), then samples were electrophoresed for 1 h at 120 V. Protein was transferred to a nitrocellulose membrane by transferring for 1 h at 100 V. The membrane was incubated in 5% non-fat milk for 1 h at room temperature, then incubated with anti-NPR1 antibody (Agrisera, Vännäs, Sweden) overnight at 4 °C. The membrane was washed three times for ten minutes in 1× PBST (0.1% Tween20), then secondary antibody was added at a ratio of 1:5000 and incubated at room temperature for 2 h. The membrane was washed as above, then incubated in Bio-Rad ECL substrate for 5 min at room temperature. X-ray film was used to capture the resulting chemiluminescence.

### 4.4. RT-qPCR

Three-week-old *A. thaliana* were sprayed with 1 mM SA or SA analogs as above, and samples were collected after 24 h. Composite samples were collected consisting of one leaf from ten biological replicates. Each leaf was of a similar size and age. Samples were frozen immediately in liquid nitrogen and crushed using a Genogrinder at 1200 RPM for 2 min. The RNA was extracted using RNAzol^®^ RT from Millipore Sigma per the manufacturer’s instructions. The RNA concentration and purity were quantified spectroscopically by measuring absorbance at 260 and 280 nm. qScript™ cDNA SuperMix from QuantaBio was used to generate cDNA from 1 μg of the extracted RNA according to the manufacturer’s instructions. PerfeCTa SYBR^®^ Green SuperMix from QuantaBio was used to perform qPCR per the manufacturer’s instructions. Relative expression levels were calculated using the double-delta Ct method. The assays were performed with ten biological replicates and six technical replicates. The primers used are listed in the table below (Table 3).

### 4.5. Preparation of SA Analog Solutions

SA and AcSA were dissolved in sterile, de-ionized water, then diluted to the necessary concentrations. All other SA analogs were diluted in 100 µL of 100% ethanol or DMSO, then diluted to their respective concentrations with sterile, de-ionized water as needed. All solutions were filter–sterilized using a 0.2 µm syringe filter prior to use.

## Figures and Tables

**Table 1:** List of mined SA analogs sorted by logP value.

Acid	Name	Molecular_Weight	LogP
3469	2,5-dihydroxybenzoic acid	1.53 × 10^2^	6.67 × 10^−1^
9338	2,6-dihydroxybenzoic acid	1.53 × 10^2^	6.67 × 10^−1^
55251260	Lithium 2,5-dihydroxybenzoate	1.60 × 10^2^	6.67 × 10^−1^
1491	2,4-Dihydroxybenzoic acid	1.53 × 10^2^	6.67 × 10^−1^
23663423	Monosodium 2,4-dihydroxybenzoate	1.76 × 10^2^	6.67 × 10^−1^
3418	Fosfosal	2.17 × 10^2^	1.1109
11812	2-Hydroxyisophthalic acid	1.80 × 10^2^	1.3557
97257	2-Hydroxyterephthalic acid	1.80 × 10^2^	1.3557
6998	Salicylaldehyde	1.22 × 10^2^	1.4218
67658	5-Fluorosalicylic acid	1.55 × 10^2^	1.4986
54675839	2,5-Dihydroxybenzoate	1.52 × 10^2^	1.5033
54712708	2,4-Dihydroxybenzoate	1.52 × 10^2^	1.5033
53629521	62TEY51RR1	3.64 × 10^2^	1.6432
16682734	Bismuth subsalicylate	3.63 × 10^2^	1.8035
8388	5-Iodosalicylic acid	2.63 × 10^2^	1.9641
72874	2-Hydroxy-4-iodobenzoic acid	2.63 × 10^2^	1.9641
4133	Methyl salicylate	1.52 × 10^2^	2.0602
8375	2’-Hydroxyacetophenone	1.36 × 10^2^	2.1286
6738	3-Methylsalicylic acid	1.51 × 10^2^	2.1672
6973	5-Methylsalicylic acid	1.51 × 10^2^	2.1672
5788	4-methylsalicylic acid	1.51 × 10^2^	2.1672
11279	2-hydroxy-6-methylbenzoic acid	1.51 × 10^2^	2.1672
164578	4-Trifluoromethylsalicylic acid	2.05 × 10^2^	2.3783
8631	3,5-Diiodosalicylic acid	3.89 × 10^2^	2.4457
8365	Ethyl salicylate	1.66 × 10^2^	2.767
54683201	Copper disalicylate	3.38 × 10^2^	2.9625
54684589	Magnesium salicylate	2.99 × 10^2^	2.965
64738	Magnesium salicylate	2.99 × 10^2^	2.965
1.02E+08	Magan	2.99 × 10^2^	2.965
517068	Calcium salicylate	3.14 × 10^2^	2.965
54684600	Calcium disalicylate	3.14 × 10^2^	2.965
1.32E+08	Magnesium salicylate	3.17 × 10^2^	3.1257
201887	2-Hydroxy-3-isopropylbenzoic acid	1.79 × 10^2^	3.5808
5282387	Magnesium salicylate tetrahydrate	3.71 × 10^2^	3.6078
54708862	Magnesium salicylate tetrahydrate	3.71 × 10^2^	3.6078
133124	Whitfield’s ointment	2.58 × 10^2^	3.7803
6873	Isobutyl salicylate	1.94 × 10^2^	4.1806
16330	Butyl salicylate	1.94 × 10^2^	4.1806
50216	Prenyl salicylate	2.06 × 10^2^	4.276
16299	Amyl salicylate	2.08 × 10^2^	4.8874
6437473	trans-2-Hexenyl salicylate	2.20 × 10^2^	4.9828
5371102	cis-3-Hexenyl salicylate	2.20 × 10^2^	4.9828
103379	Benzoic acid, 2-hydroxy-, (3Z)-3-hexenyl ester	2.20 × 10^2^	4.9828
6021887	3-Hexenyl salicylate	2.20 × 10^2^	4.9828
22629	Hexylsalicylate	2.22 × 10^2^	5.5942
153705	3-Hexylsalicylic acid	2.21 × 10^2^	5.7012
196549	Tcp (antiseptic)	5.56 × 10^2^	6.2422

**Table 2:** List of Tested SA Analogs with Chemical Structures.

ID	Name	Abbv.	Structure	Formula	Mol. Weight
1	Sodium Salicylate	NaSA	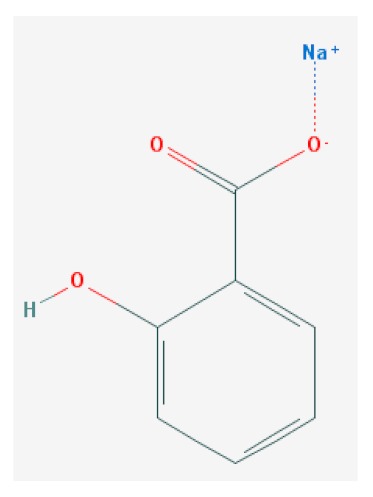	C_7_H_5_NaO_3_	160.104 g/mol
2	4-Hydroxybenzoic Acid	4-HBA	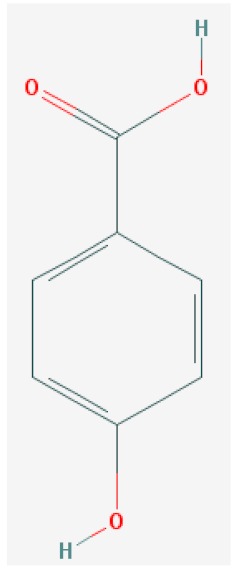	C_7_H_6_O_3_	138.122 g/mol
3	Acetylsalicylic Acid	AcSA	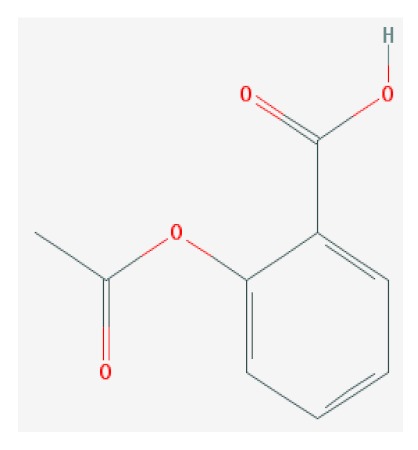	C_9_H_8_O_4_	180.159 g/mol
4	5-Aminosalicylic Acid	5-AminoSA	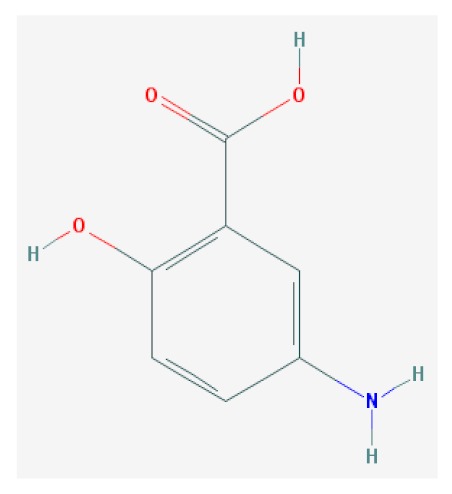	C_7_H_7_NO_3_	183.137 g/mol
5	Ethyl Salicylate	EtSA	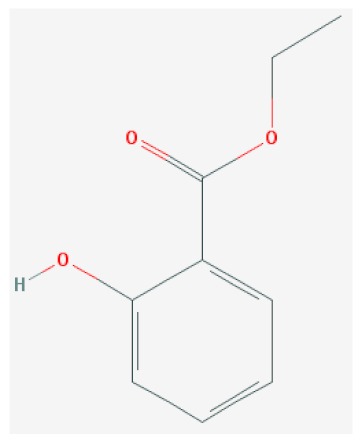	C_9_H_10_O_3_	166.167 g/mol
6	2,5-Dihydroxybenzoic Acid	2,5-DHBA	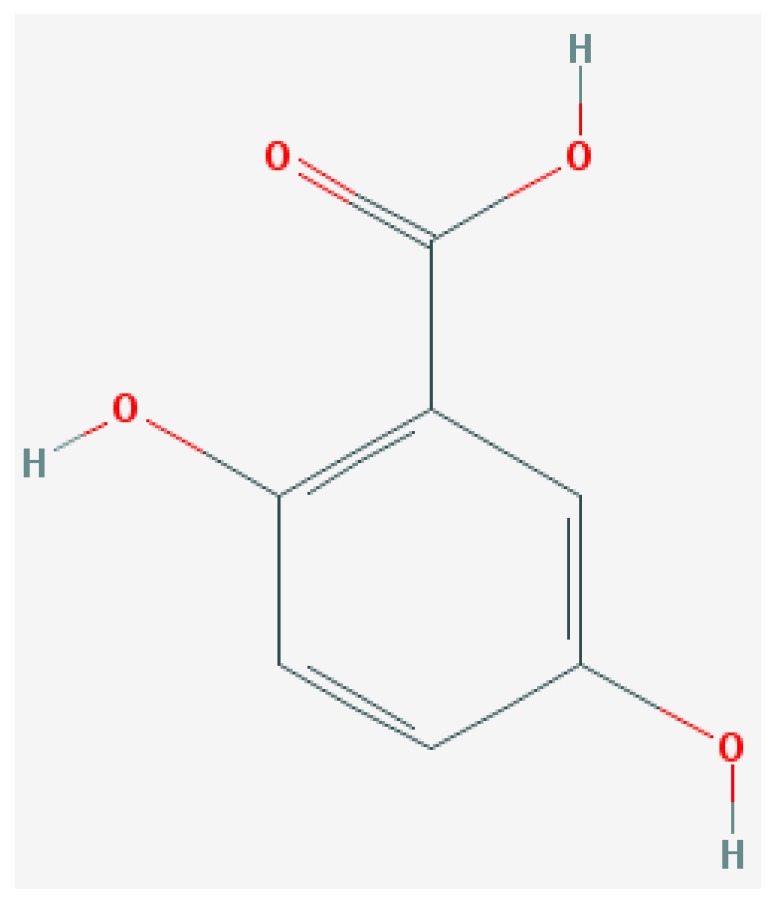	C_7_H_6_O_4_	154.121 g/mol
7	5-Methylsalicylic Acid	5-MeSA	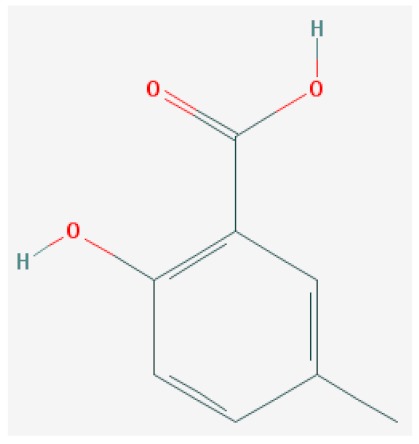	C_8_H_8_O_3_	152.149 g/mol
8	5-Iodosalicylic Acid	5-I-SA	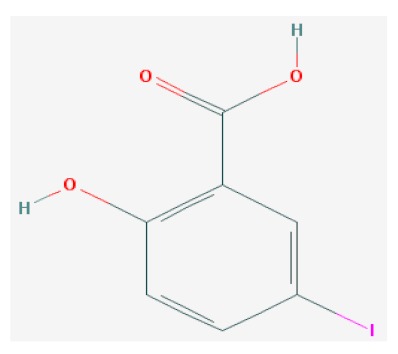	C_7_H_5_IO_3_	264.018 g/mol
9	5-Fluoro-2-Hydroxybenzoic Acid	5-F-2-HBA	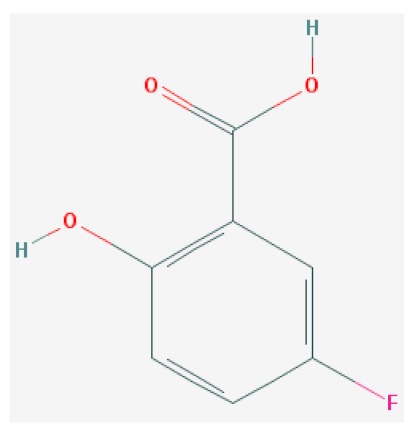	C_7_H_5_FO_3_	156.112 g/mol
10	2,4-Dihydroxybenzoic Acid	2,4-DHBA	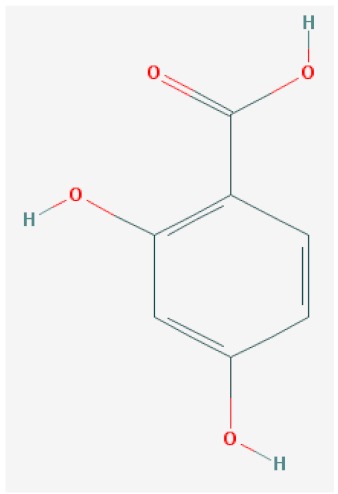	C_7_H_6_O_4_	154.121 g/mol
11	2-Hydroxyterephthalic Acid	2-HTPA	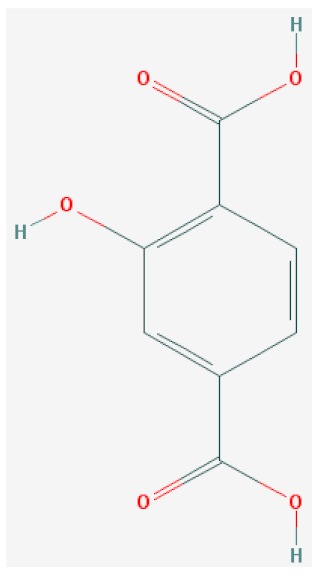	C_8_H_6_O_5_	182.131 g/mol

List of tested SA analogs, including their chemical formulas, abbreviations, molecular weights, and structures [43].

**Table 3:** List of RT-qPCR Primer Sequences.

Name	Sequence
*UBQ5* forward RT	TCTCCGTGGTGGTGCTAAG
*UBQ5* reverse RT	GAACCTTTCCAGATCCATCG
*PR1* forward RT	GCAACTGCAGACTCATACAC
*PR1* reverse RT	GTTGTAGTTAGCCTTCTCGC
